# Policy Series/ESPO and Social Research, Policy, and Practice Section Symposium: It’s a Practice, Not an End State: Centering Equity in Gerontological Research and Policy

**DOI:** 10.1093/geroni/igab046.1236

**Published:** 2021-12-17

**Authors:** Sarah Dys, Claire Pendergrast

## Abstract

Social, economic, and health inequities shape the experience of aging, reflecting a landscape of unequal resources, opportunities, and stressors that accumulate over the life-course. These inequities are not accidental, but rather reflect systems of power that act through institutions, policies, and people to simultaneously privilege some groups and disadvantage others based on socially constructed categories. These systems include, but are not limited to, racism, ageism, and capitalism. The unequal and unjust distribution of resources and opportunities over the lifespan results in health, social, and economic disparities in older adulthood. For example, Black older adults are at higher risk of experiencing chronic disease burden and shorter life expectancy than white older adults due to greater economic disinvestment, interpersonal and systemic racial discrimination, and lower health services access over the life course. This symposium features three leading scholars whose work centers racial and health equity in later life. The symposium will engage with issues related to long-term services and supports infrastructure, community-engaged and culturally relevant programs and education, and research activities (e.g., recruitment, study design, grant writing, dissemination). Panelists will also discuss their research agendas and recent scholarship, career trajectories, insights, and practices. We hope symposium attendees will identify opportunities and strategies for focusing on elimination of health disparities across the life-course in their own work. We believe this symposium can serve as an opportunity for SRPP members and emerging scholars and practitioners to center equity, highlight intersectionality, and amplify our colleagues at the forefront of addressing inequity through their work.

